# Medial Pontomedullary Stroke Mimicking Severe Bell’s Palsy: A Case Report

**DOI:** 10.5811/cpcem.2020.5.46965

**Published:** 2020-06-20

**Authors:** Benjamin Boodaie, Manish Amin, Katayoun Sabetian, Daniel Quesada, Tyler Torrico

**Affiliations:** * Kern Medical, Department of Emergency Medicine, Bakersfield, California; † Kern Medical, Department of Neurology, Bakersfield, California; ‡ Ross University School of Medicine, Department of Medicine, Miramar, Florida

**Keywords:** Bell’s Palsy, pontine stroke, MRI

## Abstract

**Introduction:**

Patients with acute unilateral upper and lower facial palsy frequently present to the emergency department fearing they have had a stroke, but many cases are benign Bell’s palsy.

**Case Report:**

We present a rare case of a medial pontomedullary junction stroke causing upper and lower hemifacial paralysis associated with severe dysphagia and contralateral face and arm numbness.

**Conclusion:**

Although rare, pontine infarct must be considered in patients who present with both upper and lower facial weakness. Unusual neurologic symptoms (namely diplopia, vertigo, or dysphagia) and signs (namely gaze palsy, nystagmus, or contralateral motor or sensory deficits) should prompt evaluation for stroke.

## INTRODUCTION

Acute facial palsy is a commonly encountered complaint in the emergency department (ED) setting. Bell’s palsy represents approximately half of such cases.^1^,^2^ Bell’s palsy is defined as an idiopathic peripheral facial nerve palsy, which is classically although controversially attributed to herpes simplex virus (HSV) infection.^3^ Bell’s palsy is a diagnosis of exclusion. The differential diagnosis includes herpes zoster (Ramsey-Hunt syndrome), otitis media, Guillain-Barré syndrome, Lyme disease, sarcoidosis, amyloidosis, parotid gland tumor, temporal bone biopsy, trauma, acoustic neuroma, central nervous system (CNS) infection, and stroke.

The presentation of a peripheral type facial paresis with weakness of both upper and lower musculature is generally reassuring that the patient has not had a stroke. However, a rare but important subset of patients who present with complete hemifacial paresis has a stroke at the level of the lower pons.^4^ In fact, one percent of all new facial paralysis cases represent a pontine stroke.^5^ In a surveillance study of almost 44,000 diagnoses of Bell’s palsy within California EDs between 2005 and 2011, 0.8% received an alternative diagnosis after 90-day follow-up, of which 30% was found to be secondary to ischemic stroke or intracranial hemorrhage.^4^ By these numbers, approximately one in 400 ED diagnoses of Bell’s palsy may be missed diagnoses of stroke.

In this report, we describe a rare presentation of medial pontomedullary junction (MPMJ) infarct that presented as unilateral peripheral type facial paresis, severe dysphagia, and contralateral face and arm numbness. We also review the literature on strokes causing peripheral facial nerve palsy and discuss important clinical flags that should raise suspicion for such pathology.

## CASE REPORT

A 63-year-old Hispanic male with untreated hypertension presented to the ED with a chief complaint of facial droop. Three hours prior to presentation, the patient noticed his face “drooping to the right,” with associated left-sided headache, left face and body numbness, chest pain radiating to his left arm, and shortness of breath. Upon arrival to the ED, the patient’s symptoms had self-resolved and facial asymmetry was absent on exam. Vitals were notable for a blood pressure of 212/123 millimeters of mercury. The National Institutes of Health Stroke Scale in the ED was calculated as one for mild, left-sided sensory deficit, which soon resolved. Computed tomography (CT) and magnetic resonance imaging (MRI) of the brain showed chronic periventricular ischemic changes but were negative for acute ischemia or hemorrhage. Laboratory studies were notable for an initial troponin of 0.09 nanograms per milliliter (ng/mL) (reference range: <0.05 ng/mL), which rose to 0.12 ng/mL four hours later. The patient was admitted for suspected acute coronary syndrome; he was started on dual antiplatelet therapy, enoxaparin, and antihypertensive therapy.

On day two of admission, the patient developed severe left upper and lower facial weakness and inability to swallow. Physical exam showed a complete paralysis of the left upper and lower face resembling severe Bell’s palsy with mild to moderate dysarthria, and decreased sensation to pinprick and cold temperature of the right face and arm. Otherwise, the patient had no upper or lower extremity motor weakness, normal extraocular movements, symmetric pupils and palatal elevation, no ptosis, and no hoarseness. A tentative diagnosis of severe Bell’s palsy was made, although stroke remained on the differential diagnosis. Repeat MRI was ultimately completed on day four of admission (approximately 2.5 days after onset of in-hospital symptoms) and was notable for a 1.5-centimeter area of increased signal intensity on diffusion-weighted imaging located at the left MPMJ consistent with an acute infarction (Image). CT angiography of the head and neck was negative for vertebrobasilar stenosis or dissection. Left heart catheterization showed mild-moderate multivessel coronary artery disease. Echocardiography showed an ejection fraction of 30%, which was believed to be secondary to long-standing uncontrolled hypertension.

The patient failed a swallow evaluation by speech therapy; the swallow response (when present) was severely weak and uncoordinated with delayed initiation and profoundly reduced laryngeal elevation, epiglottis inversion, pharyngeal constriction, and upper esophageal opening. Since significant aspiration was observed across all consistencies, the patient received a gastrostomy tube. Physical therapy evaluation revealed a new balance deficit requiring a front wheel walker.

## DISCUSSION

In patients with unilateral facial palsy, if the facial weakness is limited to the lower face, stroke is an important diagnosis to consider. This is because the frontalis muscle receives bilateral supranuclear innervation and, thus, strokes that occur above the facial nucleus (i.e., cortical, subcortical, and upper pontine strokes) will spare the upper facial muscles. Strokes occurring at the level of the lower pons that involve the facial motor nucleus or the infranuclear facial nerve can result in complete facial paralysis on the ipsilateral side and thus can mimic Bell’s palsy. Albeit rare, clinicians must be vigilant for such presentations, which can have significant morbidity if misdiagnosed.

**CPC-EM Capsule**

What do we already know about this clinical entity?

In rare cases, pontine stroke can present with both upper and lower unilateral facial weakness and mimic disease of the peripheral facial nerve (e.g., Belly’s palsy).

What makes this presentation of disease reportable?

This patient suffered from a rare stroke (reported only one other time) that led to upper and lower hemifacial paralysis, dysphagia, and contralateral face and arm numbness.

What is the major learning point?

All patients with a peripheral-type facial paralysis should be evaluated with a full neurologic exam and review of systems to evaluate for stroke.

How might this improve emergency medicine practice?

Our report highlights this easy-to-miss but potentially debilitating presentation of stroke and the specific neurologic signs and symptoms to look out for.

Pontine stroke syndromes affecting the facial nerve have been well described. They include Gasperini syndrome (facial palsy and abducens nerve palsy), Foville syndrome (facial palsy, conjugate gaze paralysis, and contralateral hemiparesis), and Millard-Gubler syndrome (facial palsy and contralateral hemiparesis). The neurologic signs that accompany these syndromes can be deduced from neuroanatomy. The facial motor nucleus is located in the lower third of the pons. This nucleus gives rise to facial motor nerve roots, which pass around the abducens nerve before they emerge from the brainstem. Thus, lower pontine strokes affecting the facial nerve commonly also affect the abducens nerve and cause abducens palsy with diplopia. Similarly, involvement of the corticospinal tract within the dorsal tegmentum can cause contralateral hemiplegia; and involvement of the paramedian pontine reticular formation can cause conjugate gaze palsy. Thus, in the context of a peripheral-type facial palsy, a lateral gaze defect and/or contralateral hemiplegia can clue providers into the possibility of a pontine infarct.

The patient reviewed in our case also had a stroke-induced facial palsy, but his clinical presentation was distinct from these aforementioned syndromes. Our patient had an acute MPMJ infarct presenting as ipsilateral complete facial hemiparesis, severe dysphagia, and a contralateral face and arm numbness. To the best of our knowledge, there is but one similar case report in the literature by Yoneoka et al in 2019.^6^ And to our knowledge, there are no reported cases in the emergency medicine literature. The infarct can be attributed to a branch occlusion of the anterior inferior cerebellar artery, as speculated by Yoneoka et al, or an occlusion of a paramedian perforating artery arising from the basilar artery.^6^

Bell’s palsy is, by definition, an isolated peripheral facial nerve lesion; the presence of additional neurologic signs or symptoms, especially those associated with the above-mentioned pontine syndromes, should prompt evaluation for stroke. It should be noted, however, that a sensation of ipsilateral facial numbness in the paretic area with hypoesthesia to pinprick (possibly secondary to contiguous spread of HSV to the trigeminal nerve) is not an uncommon finding in Bell’s palsy and should not be mistaken for stroke.^7^.^8^ Stroke and Bell’s palsy can further be distinguished based on timing of onset; the manifestations of stroke tend to progress over seconds to minutes, whereas Bell’s palsy tends to progress over hours to days. It is therefore possible for a patient to wake up with either Bell’s palsy or pontine stroke. This evaluation for stroke as a mimic of Bell’s palsy should of course be coupled with an assessment for other causes of unilateral facial paralysis, especially those for which misdiagnosis can lead to significant morbidity. These include herpes zoster (scabbing or vesicles on external ear exam); parotid gland lesions (history of facial twitching/spasms or palpable mass on exam); and Lyme disease (bilateral facial palsy or concerning history leading to serologic testing).

An additional challenge in the evaluation of pontine strokes lies in the limitations of imaging. Approximately 30% of vertebrobasilar ischemic strokes are missed on initial diffusion-weighted imaging (DWI) obtained in the first 24 hours after symptoms onset.^9^ Thus, vertebrobasilar strokes cannot be ruled out by an early negative DWI. In the patient described in this report, the initial MRI was negative in the setting of resolved symptoms, and thus he initially suffered from a transient ischemic attack. Repeat imaging did not occur until approximately 2.5 days following in-hospital symptom onset, which likely improved yield. It is important for providers to be aware of the limitations of MRI in this acute window and to trust their clinical judgment if concerning neurologic signs and symptoms persist despite a negative MRI.

## CONCLUSION

Emergency physicians must remain vigilant for acute pontine strokes presenting as complete hemifacial paresis mimicking Bell’s palsy. Unusual clinical symptoms (namely diplopia, dysphagia, and vertigo) as well as abnormalities on neurologic examination apart from the facial nerve (namely gaze palsy, nystagmus, and contralateral motor or sensory deficits) should prompt evaluation for stroke. Moreover, given the unreliability of MRI in acute brainstem stroke diagnosis, emergency physicians should trust their clinical judgment even when opposed by radiographic data and consider admitting the patient for further workup.

## Figures and Tables

**Image:**
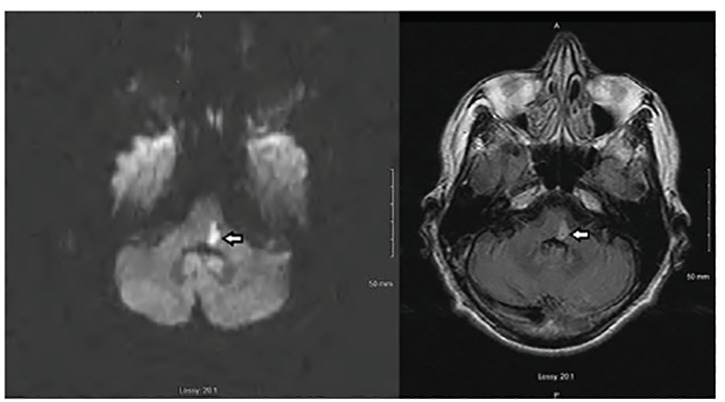
Magnetic resonance images showing left medial pontomedullary junction infarction. Axial diffusion-weighted image (left) and fluid-attenuation inversion recovery image (right) demonstrate the acute ischemic lesion. ⇦ Site of infarction.
